# Colony stimulating factor-1 in saliva in relation to age, smoking, and oral and systemic diseases

**DOI:** 10.1038/s41598-017-07698-4

**Published:** 2017-08-04

**Authors:** Ronaldo Lira-Junior, Sigvard Åkerman, Anders Gustafsson, Björn Klinge, Elisabeth A. Boström

**Affiliations:** 1 Karolinska Institutet, Department of Dental Medicine, Division of Periodontology, Stockholm, Sweden; 2 Rio de Janeiro State University, Faculty of Odontology, Department of Periodontology, Rio de Janeiro, Brazil; 3 Malmö University, Faculty of Odontology, Department of Orofacial Pain and Jaw Function, Malmö, Sweden; 4 Malmö University, Faculty of Odontology, Department of Periodontology, Malmö, Sweden

## Abstract

Colony stimulating factor (CSF)-1 is a growth factor that stimulates the survival, proliferation and differentiation of mononuclear phagocytes, which has been implicated in several inflammatory diseases. This study evaluated the possible influence of age, sex, smoking, periodontitis, caries, and several systemic conditions on salivary levels of CSF-1. Four-hundred and forty-one individuals were enrolled in this study. All participants answered a health questionnaire and underwent a comprehensive oral examination. Stimulated saliva was collected and CSF-1 levels were analysed by enzyme-linked immunosorbent assay. Salivary levels of CSF-1 were significantly increased in participants over 64 years old and in non-smoking individuals, whereas no difference was observed between men and women. Individuals having periodontitis and manifest caries had significantly higher levels of CSF-1. Participants with muscle and joint disease exhibited increased CSF-1 levels as compared to those without. Age, smoking, percentage of pockets ≥4 mm, number of manifest caries lesions, and presence of tumor were associated with CSF-1 levels. Salivary levels of CSF-1 are associated with age, smoking, periodontitis, manifest caries, and the presence of muscle and joint diseases and tumors. CSF-1 might be a promising biomarker candidate in saliva of both local and systemic conditions that needs further investigation.

## Introduction

The search for biomarkers that can accurately reflect and monitor disease states is a goal of molecular diagnostics. In this regard, saliva is emerging as a desirable body fluid that is simple, noninvasive, and inexpensive to collect. Saliva is an oral fluid whose protein composition derives mainly from salivary acinar cells, with contributions from epithelial cells shed from mucosa, blood content and tissue fluid from gingivae, and oral microorganisms^1^. It contains several biomolecules, such as DNA, RNA, proteins, metabolites, and microbiota, which can be applied to the early detection, risk assessment, diagnosis, prognosis, and monitoring of several oral and systemic infectious and immune diseases^2^. Approximately 27% of the proteins found in saliva are also found in blood^3^.

Indeed, several studies have pointed out the utility of saliva to evaluate biomarkers associated with both oral and systemic diseases^4–8^, such as higher interleukin-1β (IL-1β) levels in patients with periodontitis, and inflammatory bowel disease^8, 9^. However, most biomarker candidates so far are not solely expressed in diseased tissues, and their levels are affected by genetic, physical constitution, lifestyle and medication^10^. In fact, genetic, clinical and lifestyle factors have been shown to influence the levels of plasma biomarkers, in which as much as 56% of the biomarker variance can be attributed to non-disease factors^10^. To the best of our knowledge, no such comprehensive evaluation has been done in saliva so far. Therefore, the understanding of factors influencing immune response is necessary to comprehend inter-individual variation and its consequences on health and disease^11^.

Colony stimulating factor-1 (CSF-1), also known as macrophage colony-stimulating factor-1 (M-CSF), is a pleiotropic growth factor that stimulates the survival, proliferation and differentiation of mononuclear phagocytes^12^. CSF-1 has been implicated in inflammatory diseases, such as inflammatory bowel disease^13^ and rheumatoid arthritis^14^, and cancer^15^. CSF-1 is also implicated in periodontal disease, as highlighted by reduced alveolar bone loss when blocking the CSF-1 receptor^16^. In circulation, CSF-1 levels are associated with age in healthy individuals^17^, gender^18^, and are also influenced by weight^10^. Circulating CSF-1 levels are proposed to be a useful biomarker for lupus nephritis, as an increase in its levels predicted recurrences of nephritis before glomerular dysfunction^19^. CSF-1 levels might also be a biomarker for rheumatoid arthritis^20^, breast cancer^21^ and unstable angina^22^. We recently identified the presence of CSF-1 in saliva as a potential biomarker candidate of periodontal disease with increased levels in periodontitis compared to healthy subjects, and correlations to clinical parameters of periodontal disease severity^23^. To date, the possible impact of non-disease related factors such as age, sex﻿, and smoking on salivary CSF-1 levels are unknown. The potential association between salivary levels of CSF-1 and other oral conditions including caries, and to systemic conditions has neither been explored.

Therefore, we hypothesized that CSF-1 levels in saliva would be affected by disease- and non-disease-related covariates, which imply considering them when trying to establish cutoffs for disease diagnostics and/or monitoring. With that in mind, this study aimed to evaluate the possible influence of age, sex, smoking, periodontitis, caries, and systemic conditions on salivary levels of CSF-1, as well as to assess a normal reference range for CSF-1 in saliva.

## Results

**Figure 1:**
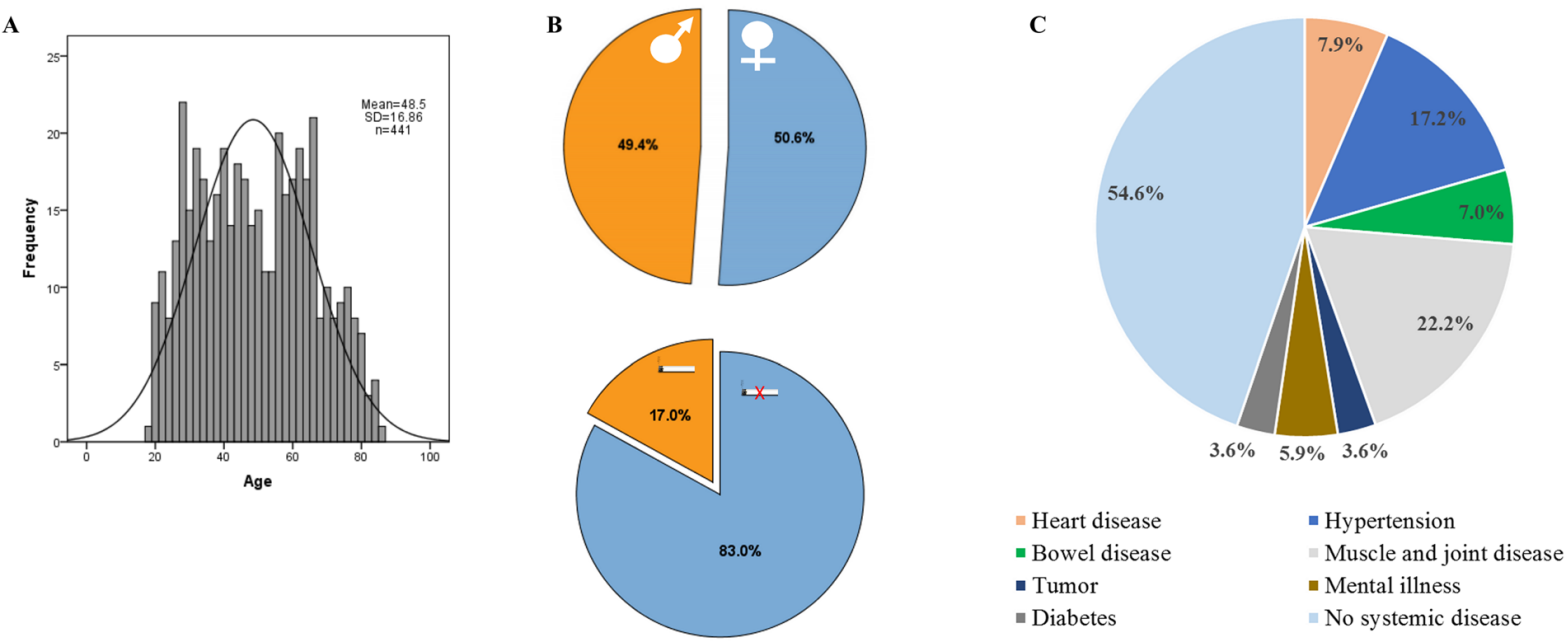
Demographics of the study population. (**A**) Histogram of age (n = 441). (**B**) Sex (223 females and 218 males) and smoking distributions (75 smokers and 366 non-smokers). (**C**) Prevalence of systemic conditions: heart disease (n = 35), hypertension (n = 76), bowel disease (n = 31), muscle and joint disease (n = 102), mental illness (n = 26), tumor (n = 16), and diabetes (n = 16).

This study analysed the influence of several disease- and non-disease-related covariates on salivary levels of CSF-1. Demographics of the cohort of 441 participants are﻿ presented in Fig. 1. We also assessed a reference range for CSF-1 in saliva of a group of non-smokers, without manifest caries, that were periodontally and systemically healthy.

### Influence of age on salivary levels of CSF-1

**Figure 2:**
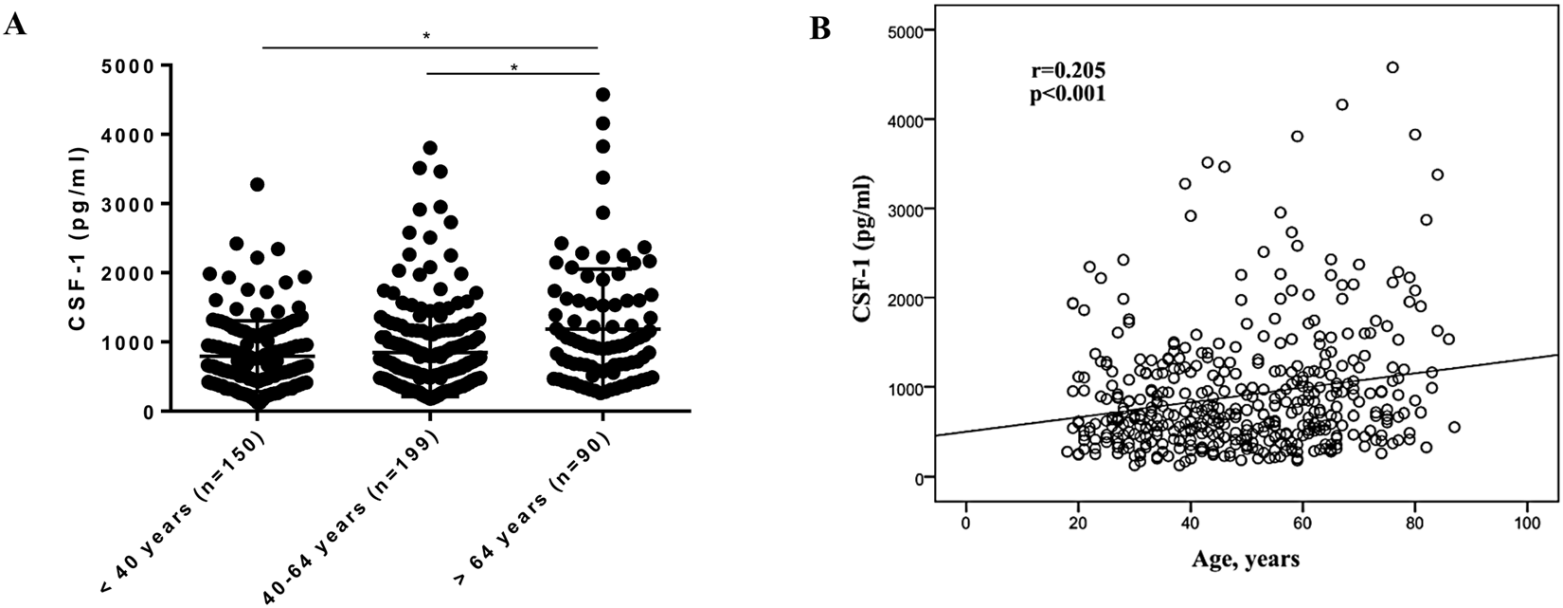
Salivary levels of CSF-1 in participants of different age groups. (**A**) CSF-1 levels in saliva of individuals <40 years (n = 150), 40–64 years (n = 199) and >64 years old (n = 90). *p-value < 0.05 (ANOVA with Bonferroni post-test). Data are presented as mean ± standard deviation. (**B**) Correlation scatterplot between CSF-1 levels and age (Pearson correlation).

Age has been associated with CSF-1 levels in blood^17^. Therefore, we compared the salivary levels of CSF-1 in three age categories (<40 years old, 40–64 years old, and >64 years old). Mean (±SD) levels of CSF-1 were 793.0 (±511.8), 848.5 (±636.8) and 1185.0 (±868.9) pg/ml for participants <40 years old, 40–64 years old, and >64 years old respectively. Participants over 64 years (n = 90) old showed significantly higher levels of CSF-1 in comparison to participants of both the age group below 40 years (n = 150) and between 40–64 years (n = 199) old (Fig. 2A). Differences remained after CSF-1 normalization for total amount of protein in saliva (Suppl. Fig.﻿ 1A). Furthermore, age was significantly correlated to CSF-1 (r = 0.205; p < 0.001, Fig. 2B). Together, these results underscore that age is associated with CSF-1 levels in saliva.

### Effects of sex and smoking on salivary levels of CSF-1

**Figure 3:**
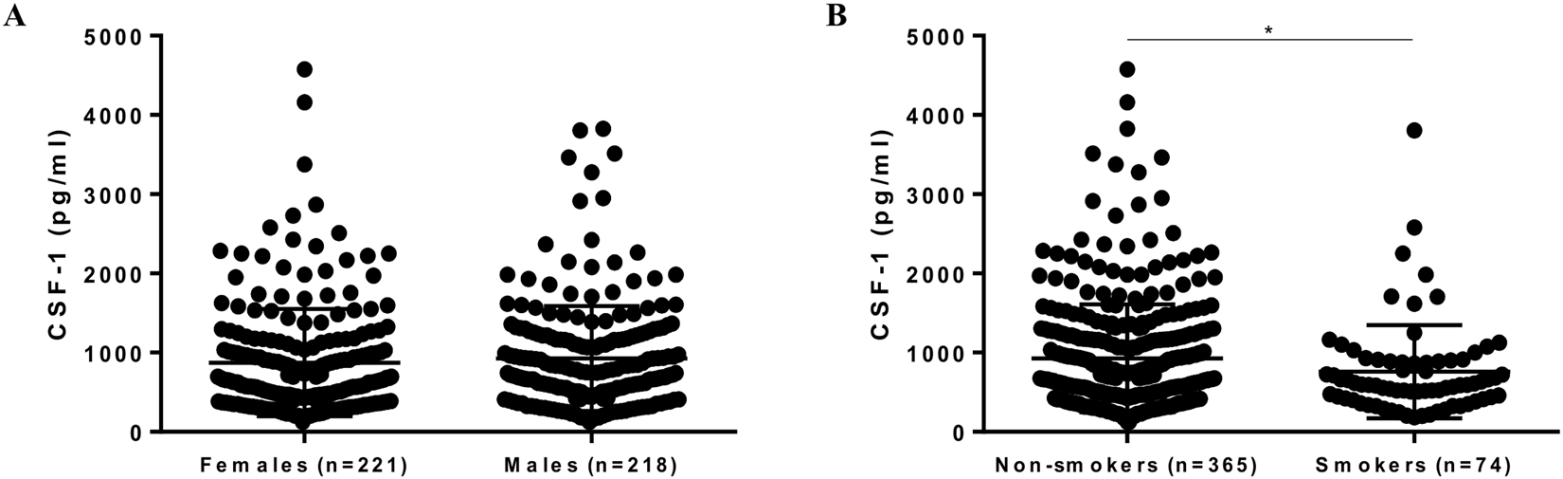
Salivary levels of CSF-1 according to sex and smoking status. (**A**) CSF-1 levels in female (n = 221) and male participants (n = 218). (**B**) CSF-1 levels in smokers (n = 365) and non-smokers (n = 74). *p-value < 0.05 (Student’s t test) Data are presented as mean ± standard deviation.

We next sought out to address the possible impact of sex and smoking on salivary CSF-1 levels. Females showed mean (±SD) CSF-1 levels of 873.7 (±677.6) pg/ml and males of 927.3 (±661.5) pg/ml. There was no significant difference in CSF-1 levels between female (n = 221) and male (n = 218) participants, both uncorrected and corrected for total amount of protein (Fig. 3A, Suppl. Fig. 1B). Regarding smoking, mean (±SD) levels of CSF-1 were 928.8 (±682.2) and 759.6 (±596.5) pg/ml for, respectively, non-smokers (n = 365) and smokers (n = 74). Smokers showed significantly lower levels of CSF-1 compared to non-smokers (Fig. 3B). This difference was lost after CSF-1 normalization for the total amount of protein (Suppl. Fig﻿. 1C).

### CSF-1 levels in saliva of participants with oral diseases

**Figure 4:**
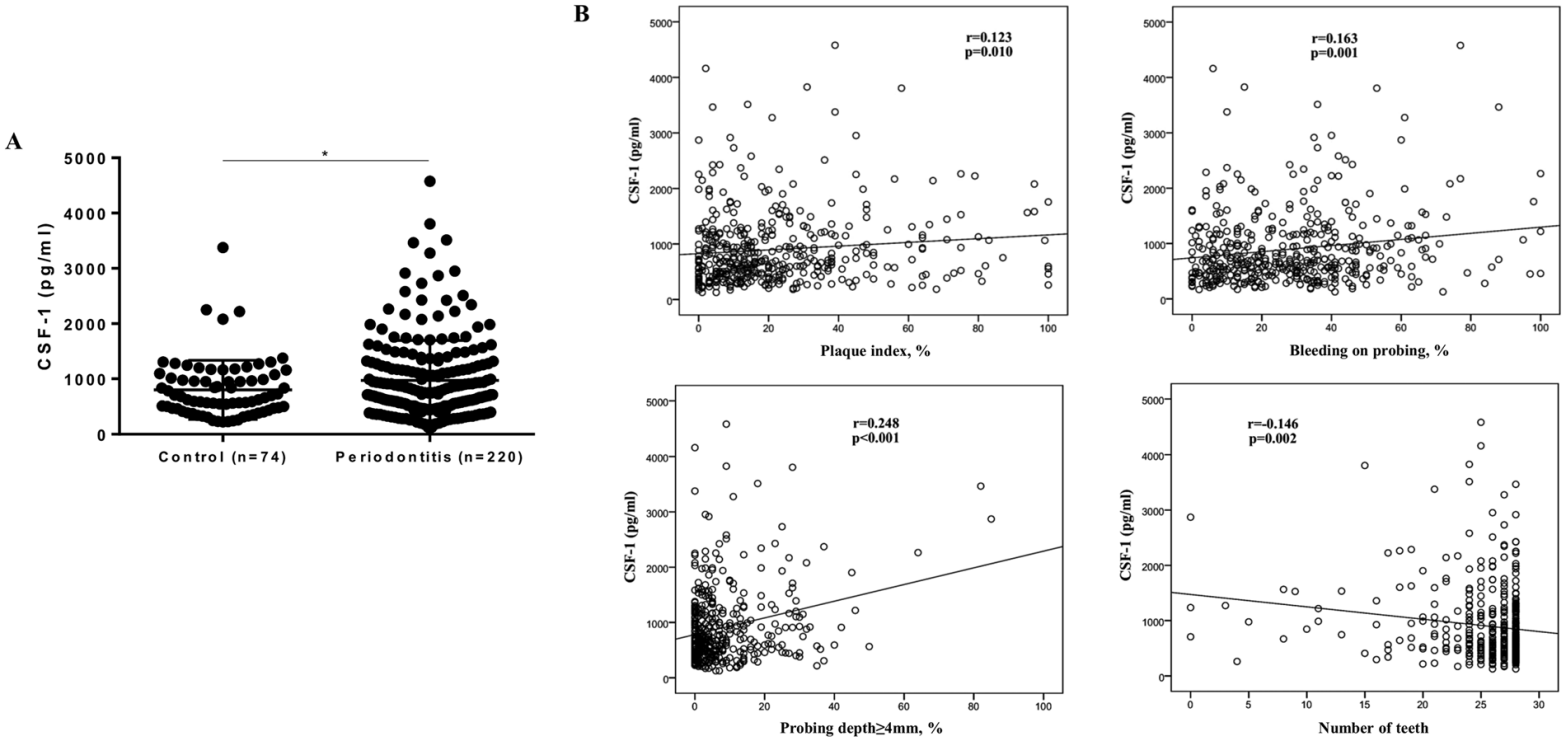
Impact of periodontal status on salivary levels of CSF-1. (**A**) CSF-1 levels in participants with periodontitis (n = 220) and controls (n = 74). *p-value < 0.05 (Student’s t test). Data are presented as mean ± standard deviation. (**B**) Correlation scatterplots between CSF-1 levels and periodontal parameters (Pearson correlation).

Based on our recent finding of higher CSF-1 levels in saliva of periodontitis compared to healthy subjects in a smaller cohort^23^, we next investigated the relation between CSF-1 levels and periodontitis in this large cohort. Participants without periodontitis (n = 74) exhibited mean (±SD) CSF-1 levels of 802.1 (±532.9) pg/ml, while participants with periodontitis (n = 220) showed mean (±SD) levels of 973.3 (±725.2) pg/ml. Salivary CSF-1 levels were significantly increased in periodontitis compared to without (Fig. 4A). This difference remained significant when restricted to patients without manifest caries (data not shown). However, differences between groups were lost when corrected for total protein (Suppl. Fig.﻿ 1D). Further, CSF-1 levels in saliva correlated significantly with PI (r = 0.123; p = 0.010), BOP (r = 0.163; p = 0.001), percentage of PD ≥4 mm (r = 0.248; p < 0.001), and number of teeth (r = −0.146; p = 0.002) (Fig. 4B).

**Figure 5:**
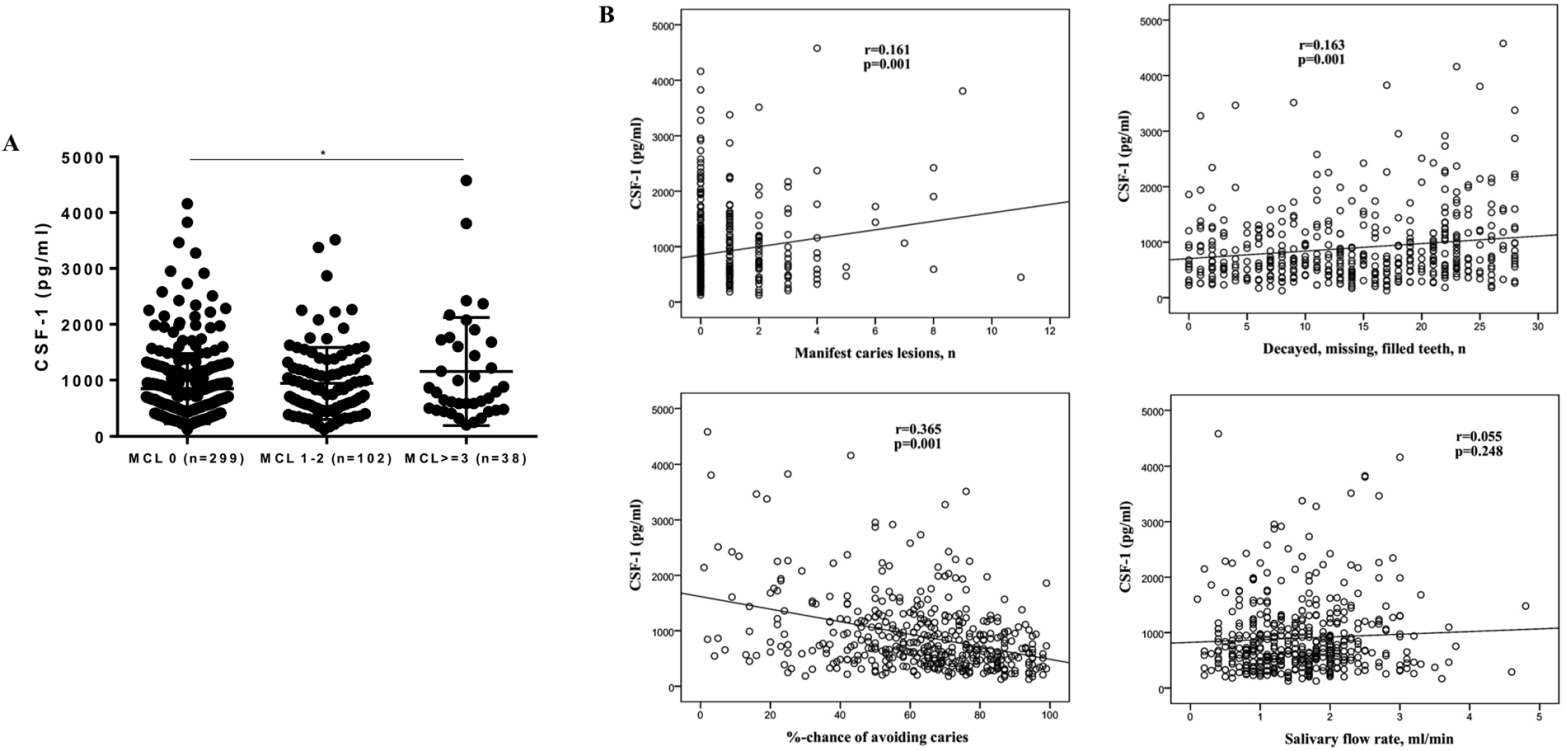
Impact of cariological status on salivary levels of CSF-1. (**A**) CSF-1 levels in participants with no manifest caries lesions (MCL) (n = 299), MCL 1-2 (n = 102) and MCL ≥3 (n = 38). *p-value < 0.05 (ANOVA with Bonferroni post-test). Data are presented as mean ± standard deviation. (**B**) Correlation scatterplots between CSF-1 levels, cariological parameters and salivary flow rate (Pearson correlation).

We next investigated the possible impact of caries on CSF-1 levels in saliva. Mean (±SD) levels of CSF-1 were, respectively, 852.2 (±626.2), 945.0 (±641.5) and 1158.2 (±964.5) pg/ml for participants with no MCL (n = 299), MCL 1–2 (n = 102) and MCL ≥3 (n = 38). Salivary CSF-1 levels were significantly higher in participants with MCL ≥3 as compared to MCL 0, whereas no difference was found between MCL 1–2 and MCL 0 or between MCL 1–2 and MCL ≥3 (Fig. 5A). The difference between groups was lost when corrected for total protein (Suppl. Fig. 1E). When participants having periodontitis were excluded, only a trend towards significant difference was observed (data not shown). Further, CSF-1 levels correlated significantly with MCL (r = 0.161; p = 0.001), DMFT (r = 0.164; p = 0.001) and caries risk (r = −0.365; p = 0.001). There was no significant correlation between CSF-1 and salivary flow rate (r = 0.055; p = 0.248) (Fig. 5B).

We also assessed correlations between the levels of CSF-1 and other inflammatory molecules previously analyzed in saliva of this cohort^4, 5^ and found significant correlations to the levels of IL-1β (r = 0.467; p < 0.001), IL-8 (r = 0.413; p < 0.001), and MMP-8 (r = 0.459; p < 0.001).

### Effects of systemic conditions on CSF-1 levels in saliva

To address the possible relation between CSF-1 levels in saliva and systemic diseases we analysed CSF-1 levels with respect to several systemic conditions. Individuals having muscle and joint diseases (n = 96) showed increased levels of CSF-1 as compared to patients not having the disease (n = 335), but the difference was lost after CSF-1 correction for total amount of protein. Participants who reported tumors (n = 16) showed a trend towards increased levels of CSF-1, however this did not reach statistical significance. There was no significant difference in mean levels of CSF-1 between participants having heart disease, hypertension, bowel disease, mental illness and diabetes and participants not having the disease (Table 1). To address the possible confounding factor of comparing participants with a particular systemic disease to participants having other systemic diseases, a reference group, which was composed by subjects free of any of the systemic conditions (n = 241), was derived. The results were roughly the same when we compared CSF-1 levels of patients having systemic conditions to the reference group (data not shown).

**Table 1:** Mean (±SD) levels of CSF-1 (pg/ml) in saliva from patients having or not systemic conditions.

Disease	No	Yes	p-value*
Heart disease (n = 34)	889.83 (±632.74)	1012.64 (±990.41)	0.482
Hypertension (n = 75)	877.56 (±632.54)	1003.74 (±808.41)	0.137
Bowel disease (n = 30)	898.93 (±664.30)	907.43 (±715.95)	0.946
Muscle and joint disease (n = 96)	863.25 (±625.82)	1026.09 (±785.37)	0.035
Tumor (n = 16)	880.24 (±637.20)	1399.46 (±1129.57)	0.087
Mental illness (n = 24)	900.90 (±663.73)	876.09 (±737.62)	0.860
Diabetes (n = 15)	893.34 (±673.23)	1070.81 (±450.20)	0.312

SD: standard deviation. *Student’s t test.

### CSF-1 reference range in saliva

In order to establish a reference range for CSF-1 in saliva, we derived a group of participants encompassing non-smokers, free of systemic diseases, without manifest caries lesions, without bone loss and periodontal pockets ≥4 mm. This group was composed by 35 subjects. Mean (±SD) CSF-1 was 763.95 (±415.55) pg/ml, ranging from 224.55 to 2218.00 pg/ml. The 95% reference range was 234.42–1862.08 pg/ml. For the normalized values, mean (±SD) was 1083.72 (±459.70) pg/mg, ranging from 405.53 to 2174.98 pg/mg. The 95% reference range was 426.57–2187.76 pg/mg. Worth noting, CSF-1 levels were detected in 100% of the saliva samples of the whole cohort (n = 439﻿).

Seeking to identify the determinants of CSF-1 levels in saliva, results from a linear regression analysis found that age, presence of tumors, percentage of PD ≥4 mm, and number of MCL were significantly associated with CSF-1 levels. Overall, these 4 variables accounted for 32.8% of the variance in CSF-1 levels (Table 2). For the normalized CSF-1, only age remained significantly associated with its levels, and it accounted for 13% of CSF-1 variance.

**Table 2:** Linear regression analysis of the association of demographic variables, oral and systemic conditions with CSF-1 (pg/ml) in saliva (n = 439).

Variables	Coefficient (β)	95% CI	p-value
Age	4.69	0.84–8.54	0.017
Smoking	−166.20	−329.24–−3.15	0.046
Tumor	417.12	90.27–743.97	0.012
Percentage of PD ≥4 mm	10.58	4.50–16.66	0.001
Manifest caries lesions	62.82	20.13–105.58	0.004

Variables included, but not retained, in the model: Sex, bleeding on probing, heart disease, hypertension, bowel disease, muscle and joint disease, mental illness, and diabetes. R^2^ = 0.328.

## Discussion

This study, to the best of our knowledge, is the first one to comprehensively evaluate how disease- and non-disease-related variables affect salivary CSF-1 levels in a relatively large number of participants. We found that age, smoking, periodontitis, caries, and the presence of muscle and joint diseases are related to altered salivary levels of CSF-1. We also established a 95% reference range for salivary CSF-1 from 234.42 to 1862.08 pg/ml in healthy individuals. The elucidation of variables that might impact CSF-1 levels in saliva increases its potential to be accurately used as a biomarker, as the variability unrelated to disease can be taken into account.

We found that age was associated with CSF-1 levels in saliva, particularly after 64 years old. We also identified, in a regression analysis, that each additional year of age is associated with an average increase of 4.69 pg/ml in CSF-1 levels. In line with the observed correlation between age and salivary CSF-1, other relations between age and inflammatory markers in saliva have been described^5, 24^. Age has an evident impact on cytokine responses, although this effect is cytokine- and/or stimuli-dependent, such as defects in the production of T-helper-related cytokines^25^. Studies investigating the relationship between age and CSF-1 in blood have been conflicting. While significant associations have been reported between CSF-1 and age in healthy individuals^17, 26–28^, another study has not found age as a significant covariate for CSF-1^10^. The significance of increased CSF-1 levels with age is unknown. However, we speculate this increase might reflect the process of inflammaging that is associated with aging and that is evident in saliva as well, as highlighted by increased markers of protein oxidation with aging in saliva^29^.

In our study, there was no significant association between sex and CSF-1 levels in saliva. In line with our finding, no significant effect of sex has been reported ﻿on blood levels of CSF-1^10, 17^. On the other hand, studies have reported higher blood levels of CSF-1 in males^18, 30^, as well as in females^31, 32^. Circulating levels of CSF-1 is further modulated by pregnancy, wherein up-regulated levels are measured in pregnant women^33^. As circulating CSF-1 levels seem to correlate with hormone concentrations (estradiol) in women, but not in men^30^, this might account for the controversies found in the literature regarding its levels. Contrary to sex, smoking exerted a significant role in CSF-1 levels in saliva. Being a smoker was associated with an average decrease of 166.20 pg/ml in CSF-1 levels. Smoking has been related to decreased levels of other inflammatory markers in saliva such as IL-8 and MMP8/TIMP1 ratio^5^. Liede *et al*. reported lower salivary MMP-8 levels in current smokers^34^. Decreased flow of gingival crevicular fluid in smokers, which would lead to a lower influx of inflammatory mediators into saliva, might be one possible reason for this decrease^35^. Lower levels of CSF-1 in serum have been found in smoking patients with long bone fractures^32^, whereas others found no significant effect of smoking on plasmatic CSF-1^10^. Together, our data indicates the need of taking age and smoking into account when relating salivary CSF-1 levels to diseases.

We reported significantly higher levels of CSF-1 in participants diagnosed with periodontitis, even when restricted to patients not having manifest caries. We also found significant correlations to clinical periodontal parameters. These results confirm, in a larger group, recent findings of increased CSF-1 levels in saliva from periodontitis patients by our group^23^. Further, CSF-1 levels correlated significantly with the levels of IL-1β, IL-8, and MMP-8, which were previously analysed in participants from this cohort^5^. This is also in line with our previous finding of a significant correlation between the levels of CSF-1 and MMP-8 in saliva^23^. Blocking of the CSF-1 receptor has been shown to reduce alveolar bone loss and number of osteoclasts in experimental periodontitis in mice^16^. Further, in experimental arthritis in mice injections of CSF-1 have been shown to exacerbate disease whereas CSF-1 blocking alleviates disease severity^14^. Along with the fact that CSF-1 stimulates RANKL-induced osteoclast formation^36^, increased salivary levels of CSF-1 might be reflective of higher osteoclastogenic potential in periodontitis.

Participants with high level of caries (MCL ≥3) exhibited higher levels of CSF-1 in saliva. Further, we found a significant correlation between salivary CSF-1 and caries risk. An association between MCL and MMP-8 has previously been reported in this cohort^37^. However, when we restricted the analysis to participants not having periodontitis, there was only a trend towards higher CSF-1 levels in subjects with manifest caries (p-value = 0.092), which suggest that the effects of caries on CSF-1 levels might partially be due to their periodontal condition. Dental pulp fibroblasts from dentinal caries sections immunostained for CSF-1 in about 38% of the teeth, while no immunostaining was observed in sections from non-carious teeth. Moreover, TNF-α-stimulate pulp fibroblasts produced CSF-1^38^. We can speculate that, at least partially, CSF-1 might leak out from the carious dentin to saliva.

Inflammatory molecules in saliva have been investigated in relation to systemic diseases and their utility as biomarkers have been demonstrated^4, 7^. Our study found increased salivary levels of CSF-1 in participants having muscle and joint diseases, which is in line with findings of higher CSF-1 in serum and urine from patients with systemic lupus erythematosus^19^. Increased expression of CSF-1 in plasma has also been reported in active as compared to quiescent rheumatoid arthritis^20^. Our study also showed that the presence of tumors was associated with CSF-1 levels. Elevated serum levels of CSF-1 have been found in patients with breast cancer, and even higher in patients with invasive cancer and concomitant lymph node metastasis^21^. Increased serum levels have also been reported in patients with colorectal cancer^39^. Our result thus suggests that assessment of CSF-1 levels in saliva might be valuable for monitoring patients with muscle and joint diseases and tumors however, further investigations are needed.

The main limitation of this study is its cross-sectional nature and, thus, no causal claim can be done. Prospective studies would be of great value to confirm hypotheses generated in this study, as well as the addition of gingival crevicular fluid. Besides, medical conditions were determined by self-report and, then the diseases were grouped together in broader categories. Therefore, we cannot rule out that particularities from each disease might influence differently on CSF-1 levels, as well as medications used to treat them and the disease status during saliva collection, such as active/quiescent and controlled/uncontrolled. We also cannot rule out that other covariates might play a role in determining salivary levels of CSF-1. Indeed, an impact of genetic factors has been reported on the variation of plasmatic CSF-1 levels^26^, thus we speculate that genetic factors might also play a role in the variation of salivary CSF-1. As saliva is a complex biologic fluid, attention to its collection method is mandatory, which can have an impact on the concentration of proteins. Generally, levels of inflammatory biomarkers are higher in unstimulated than stimulated whole saliva^40^. However, we have shown no significant difference in CSF-1 levels between unstimulated and stimulated saliva, either fasting or non-fasting^23^. We also reported that the significant differences in CSF-1 levels were lost for smoking, periodontitis, caries, and muscle and joint diseases when correcting for total amount of protein. This might be explained by the higher amount of total protein in these groups of patients, which could be a consequence of increased plasma protein leakage during the inflammatory process. Higher protein concentration in periodontitis patients has been shown, which could not be attributed to increased salivary flow rate^41, 42^. On the other hand, decreased concentration of total protein has been reported in smokers^43^.

In conclusion, this study found that age, smoking, periodontitis, manifest caries, and the presence of tumors are associated with the salivary levels of CSF-1. Furthermore, these results showed increased CSF-1 levels in patients reporting muscle and joint diseases.

## Methods

### Study sample

This cross-sectional study enrolled 441 individuals (48.5 [±16.8] years; 50.6% women) living in Skåne, Sweden, who were orally examined and provided a saliva sample. Study protocol was approved by the Research Ethics Committee at the Lund University, Sweden, and all participants gave their written informed consent. All methods were performed in accordance with the relevant guidelines and regulations. Participants were asked to answer a questionnaire regarding oral health, and data concerning age, sex, smoking habits (including use of Swedish snus), presence of diseases, and use of medication were collected. Smoking was recording as yes or no. Diseases were self-reported and grouped as heart disease, hypertension, bowel disease, muscle and joint disease, mental illness, tumor, and diabetes. Details of the sample selection and the questionnaire were described previously^44^.

### Clinical examination

90.5% of the clinical examinations were performed by four dentists employed at the Department of Oral Diagnostics, Faculty of Odontology, Malmö University. Periodontal parameters included visible plaque index (PI), bleeding on probing (BOP) and probing depth (PD), all recorded in four sites on each tooth using a periodontal probe (PCPUNC15, Hu-Friedy, IL, USA). In addition, digital bitewings and panoramic radiographs were taken for evaluation of alveolar bone loss. Periodontitis was defined as the presence of PD ≥4 mm and BOP >20%. Participants having no PD ≥4 mm and bone loss not exceeding 1/3 of the root length were selected as controls.

Cariological parameters included DMFT (decayed, missing, filled teeth), number of manifest caries lesions (MCL), which encompassed lesions involving dentin, as seen on bitewing radiographs and cavitated lesions on other surfaces, and caries risk. Number of MCL was grouped as previously described: MCL 0: no MCL, MCL 1–2: 1 or 2 MCLs and MCL ≥3: 3 or more MCLs^37^. Caries risk was evaluated using the Cariogram, showing the %-chance of avoiding caries^45^.

### Saliva collection

Stimulated saliva was collected during 5 minutes chewing 0.5 g of paraffin in a graded tube. Secretion rate (ml/min) was determined after excluding the foam. Samples were immediately frozen at −20 °C until processing. Saliva samples were then centrifuged and supernatants aliquoted and stored at −80 °C. Each aliquot was used only once for biomarker analysis. For the analysis of CSF-1, 439 samples were included.

### CSF-1 immunoassay

Levels of CSF-1 in saliva were determined using a commercial enzyme-linked immunosorbent assay (ELISA) according to the manufacturer’s instructions (R&D Systems, Minneapolis, MN, USA). Samples were thawed on ice and centrifuged at 10000 rpm for 5 min prior to analysis. The detection limit of the assay was 78.1 pg/ml and the sensitivity 11.2 pg/ml. Readings were made at 450 nm with wavelength correction set to 540 nm to subtract background using a microplate spectrophotometer (SpectraMAX 340, Sunnyvale, CA, USA). Total amount of protein was determined by the Bradford assay to enable normalization of CSF-1 levels to total salivary protein.

### Statistical analysis

Data analyses were performed using Statistical Package for Social Sciences (SPSS) version 20 (IBM Corporation, Armonk, NY, USA). Continuous variables are presented as mean and standard deviation (SD) and categorical variables as frequencies. Group comparisons were performed with Student’s t-test or ANOVA with Bonferroni post-test. Correlations between CSF-1 levels and other variables were determined by Pearson correlation coefficient. A multiple linear regression was done with CSF-1 as dependent variable and age, sex, smoking, bleeding on probing, percentage of PD ≥4 mm, number of MCL, and the systemic conditions as independent variables. Statistical significance was set at *P* ≤ 0.05.

In order to establish a reference range for CSF-1 in saliva, a 95% reference range was calculated as mean ± (1.96 × SD). For that purpose, CSF-1 values were log-transformed in order to achieve a normal distribution, and then the anti-log was calculated to exhibit the reference range in pg/ml.

## Electronic supplementary material

Supplementary  Information 
