# Supplementary figures and images for: Colony stimulating factor-1 in saliva in relation to age, smoking, and oral and systemic diseases
Source: Sci Rep. 2017 Aug 4;7:7280. doi: 10.1038/s41598-017-07698-4 (PMC5544729; doi:10.1038/s41598-017-07698-4)

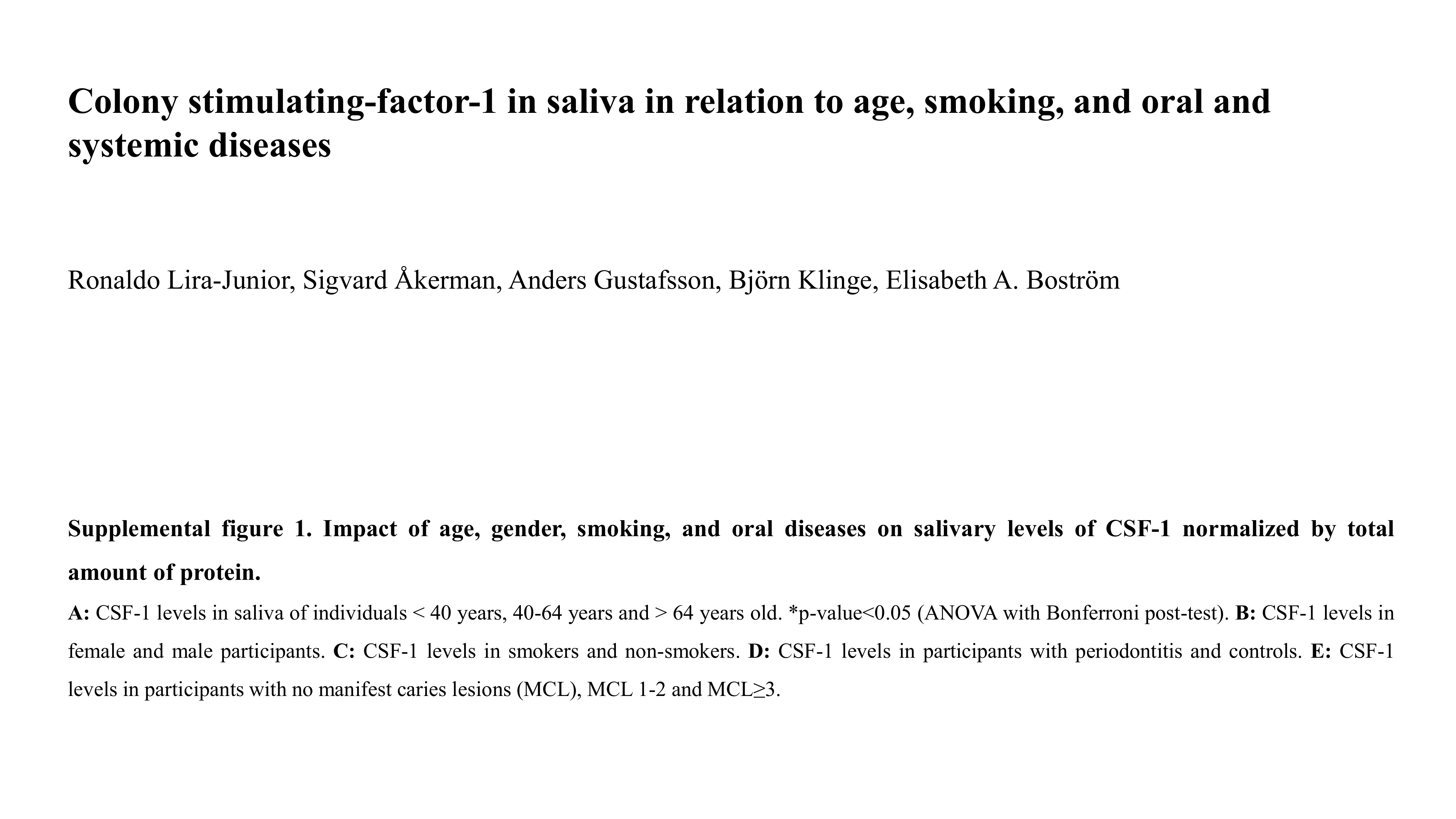

Supplement: Supplementary file 1 — Supplementary Information [file 41598_2017_7698_MOESM1_ESM.tif]
